# Knowledge, attitudes and behaviours of women regarding breast and cervical cancer in Malatya, Turkey

**DOI:** 10.1371/journal.pone.0188571

**Published:** 2017-11-28

**Authors:** Serdar Deniz, Burak Kurt, Ayşe Ferdane Oğuzöncül, Ersin Nazlıcan, Muhsin Akbaba, Tufan Nayir

**Affiliations:** 1 Malatya Health Directorate, Malatya, Turkey; 2 Cukurova University Faculty of Medicine, Department of Public Health, Adana, Turkey; 3 Firat University Faculty of Medicine, Department of Public Health, Elazığ, Turkey; 4 Ministry of Health of Turkey Republic, Ankara, Turkey; Rudjer Boskovic Institute, CROATIA

## Abstract

The aim of this study is to gather information about the knowledge, attitudes and behaviours of women regarding breast and cervical cancer, to increase the knowledge level of the relevant age group and to begin including the relevant age group in screening programs. This cross-sectional study is composed of 6910 women aged 30–69 years in Malatya, Turkey. The study aimed to reach 1782 women with a survey, and all of them were reached. The questionnaire form was completed with face-to-face interview. For statistical analysis, data were evaluated as number and percentage distributions. The average age of the women was 45.6±11.4. Nearly half of the women (46.4%) did not know that scans for early detection of breast and cervical cancers were free. Only 22.2% of women knew that breast cancer could be diagnosed early by mammography. 72.7% had never received a mammogram. One third (31.6%) of women did not know it was possible to recognize cervical cancer early, and two thirds (64.5%) of women had not received a Pap smear test. It has been determined that Turkish women do not have adequate knowledge about cancer diagnosis, early cancer diagnosis, and screening.

## Introduction

Cancer, which is characterized by genetic changes in and the uncontrolled proliferation of cells, is the second most common cause of death [1]. According to GLOBOCAN 2012 data, breast cancer is the second most common cancer in the world, and by far the most frequent cancer among women, with an estimated 1.67 million new cancer cases diagnosed in 2012 (25% of all cancers). Breast cancer ranks as the second cause of death from cancer overall, after lung cancer, and while it is the most frequent cause of cancer death in women in less developed regions, it is now the second cause of cancer death in more developed regions [2–4]. Cervical cancer has been determined to be the fourth most frequently observed cancer type with 528,000 new cases and 266,000 deaths expected in females in 2012. Cervical cancer is an important health problem in the world; its incidence and mortality rate are reported to be 7.9% and 7.5%, respectively [2,3]. The 5-year survival rate in patients with cervical cancer is less than 50% in developing countries and less than 66% in developed countries [5].

The prevalence of cancer in Turkey and is similar to that in other developing countries. In Turkey, breast cancer is the in the first place with 45.1 incidence per one hundred thousand, and cervical cancer is in ninth place with 7.1 incidence per one hundred thousand [6].

Certain risk factors play an important role in breast cancer development. These include breast cancer in first-degree relatives, advanced age, early menarche, and late menopause [7]. Studies that focus on the early diagnosis and treatment of breast cancer have not yet achieved their goals. Consequently, it is recognized that even among young people, knowledge about breast cancer is inadequate [8]. The inadequacy of knowledge and practice make early diagnosis and effective treatment difficult [9].

The risk factors for cervical cancer are early age at first sexual intercourse (<16 years), number of sexual partners, smoking, race, parity, and low socioeconomic status. In a study conducted between 2006 and 2008, 184 patients who underwent surgical treatment for endometrial, ovarian, and cervical cancer were evaluated retrospectively. In 33.3% of the women, the genital hygiene that is important for the prevention of cervical cancer was found to be inadequate; 40% of the women were characterized by low socioeconomic status; 66.7% had had at least one miscarriage; 33.3% had had curettage twice; and 80% did not regularly receive gynaecological testing [10].

In Turkey, cervical cancer screening starting at 30 years old and ending at 65 years old is being done as it will be done in women every 5 years. In addition, a breast cancer scan is done at the age of 40 years and ends at the age of 69 to be repeated every 2 years. It is the responsibility of the Noncommunicable Diseases, Programs and Cancer Unit in the Community Health Directorate’s offices to coordinate, register, monitor and report these screening studies to the Ministry of Health. In breast cancer screening, two mammograms are taken from two different directions for both breasts. Breast examination is also recommended. In order to achieve the goal of screening to reduce breast cancer mortality, more than 70% of the target population must be screened. HPV test or Pap smear test, which will be applied every five years, is used in cervical cancer screenings. It is known that the likelihood of cervical cancer occurring within five years is very low if the HPV test result is negative. Pap smear test is a cytological screening test. It is able to detect cervical lesions that have not yet symptoms [11].

However, according to a report published by the Turkish Statistical Institute (2012), fewer than 40% of women aged 45 to 65 have ever had a mammogram and around 30% of women aged 25 to 65 have ever had a PAP smear. Hence, a better understanding of psychosocial and demographic factors associated with screening is needed in this context [12].

Health professionals, who are in constant communication with the community, should provide accurate health information by counselling patients about the prevention of gynaecological cancer [13]. Patients play important roles in screening participation by knowing the symptoms of the illness, accepting the dangers of the illness, understanding that they could become sick, being treated (in the event of illness) according to the screening results, and believing that the results will be evaluated in a meaningful way [14].

The aim of this study is to gather information about the knowledge, attitudes and behaviours of women regarding breast and cervical cancer in order to increase the knowledge level of the relevant age group and to begin including them in screening programs.

## Materials and methods

This descriptive study is composed of 6910 women aged 30–69 years in the Akçadağ district of Malatya, Turkey. Using the Epi Info program (in which the expected frequency was selected to reach the maximum sample size of 50%), the 95% confidence interval was calculated based on 2% error, and the result was 1782. 1782 out of 6910 women were selected by systematic sampling method. Before starting the survey, 20 women were pre-tested in the same area. Non-working questions were corrected by this way. These women had not been included in statistical analysis. After this, we succeeded in reaching 100% of selected participants. Survey form developed by the researchers was used to collect the data for this study. The survey was conducted between October 2015 and February 2016 with face to face interview at participants’ home. The questionnaire, which had been used as a data collection tool contained 62 questions and consisted of three parts.

The first part of the questionnaire asked about sociodemographic status, health insurance status, number of births and number of living children, age of first menstruation, history of breast and cervical cancer in relatives, and smoking status.

The second part of the questionnaire asked about knowledge, attitudes, behaviours and diagnosis status in relation to breast cancer.

The third part of the questionnaire asked about knowledge, attitudes, behaviours and diagnosis status in relation to cervical cancer.

Before initiating this study, approval was obtained from the hospital where the study was conducted and from the Ethics Committee of Firat University, Faculty of Medicine (Meeting no.18/Decision no.4). All participants were informed by the researcher about the aims of the study, and written informed consent was obtained prior to participation. We told the participants that they could withdraw from the study at any time and that we would keep all information strictly confidential.

The SPSS 19.0 package was used for statistical analysis. Descriptive data were evaluated as number and percentage distributions. The averages are given with standard deviations.

## Results

A total of 1782 women participated in the survey. The average age of the women was 45.6±11.4, and the age distribution was between 30 and 69. More than half of the women (51.3%) were in the 30–44 year-old age group; three in four (75.9%) had education levels of elementary school or lower, and the majority (88.7%) were married. The majority of women (67.8%) were in the middle-income group, had social security (94.2%) and were not smoking (85.9%) (Table 1).

**Table 1:** Socio-demographic characteristics of women.

Characteristics		N	%
Age			
	30–34	342	19.2
	35–39	296	16.6
	40–44	276	15.5
	45–49	223	12.5
	50–54	206	11.6
	55–59	150	8.4
	60–64	145	8.1
	65–69	144	8.1
Educational status			
	Illiterate	335	18.8
	Literate	139	7.8
	Primary school	879	49.3
	Secondary school	168	9.4
	High school	203	11.4
	University	58	3.3
Marital status			
	Married	1579	88.7
	Single	52	2.9
	Widowed	129	7.2
	Divorced	22	1.2
Economic status			
	Low	208	11.7
	Middle	1208	67.8
	High	366	20.5
Social security			
	Have	1678	94.2
	Have not	104	5.8
Smoking status			
	Never	1530	85.9
	Quit	123	6.9
	Not every day but sometimes	27	1.5
	Every day at least once	102	5.7

The majority of women (83.9%) had been married between the ages of 15–24 and had given birth to their first child during that same age range (82.4%). The majority (92.9%) of women had children, and nearly half of them (42.7%) had 3–4 children. Regarding the use of contraception, it was determined that 28.7% used modern methods and that 53.2% of women did not use any method of birth control (Table 2).

**Table 2:** Fertility and reproductive health status of women.

Status		N	%
Age of marriage			
	10–14	75	4.3
	15–19	931	53.8
	20–24	521	30.1
	25–29	145	8.4
	30 and higher	58	3.4
Age at first childbirth			
	10–14	8	0.5
	15–19	654	39.7
	20–24	704	42.7
	25–29	216	13.1
	30 and higher	66	4.0
Having children			
	Yes	1656	92.9
	No	126	7.1
Number of children			
	0	126	7.1
	1–2	503	28.2
	3–4	761	42.7
	5–6	296	16.6
	7 and higher	96	5.4
Contraception used			
	Withdrawal method	321	18.0
	Intrauterine device	222	12.5
	Condom	137	7.7
	Vasectomy	83	4.7
	Pills	44	2.5
	Injection	24	1.3
	Calendar method	2	0.1
	None	949	53.2

Nearly half of the women (46.4%) did not know that screening for early detection of breast cancer and cervical cancer was free. A total of 11.2% of women had a family member with breast cancer, 4.2% with cervical cancer, and 26.4% with other types of cancer. Nearly one quarter (23.9%) of women did not think it was possible to detect breast cancer early. Only 22.2% of women knew that breast cancer could be diagnosed early by mammography, while 25.3% knew about self-examination and 37.8% knew about physician examination. Nearly half of the women (49.0%) did not perform breast self-examination, 67.2% had not received a physician’s examination, and 62.0% had never received a mammogram. Only 8.2% of women knew (correctly) that mammography should be performed once every two years after age 40, which is the Turkish standard (Table 3).

**Table 3:** Knowledge and practices regarding breast cancer.

Question		N	%
Do you know that scans for early detection of breast cancer and cervical cancer are free?			
	Yes	956	53.6
	No	826	46.4
Do you have a family member who has breast cancer?			
	Yes	199	11.2
	No	1583	88.8
Do you have a family member who has cervical cancer?			
	Yes	75	4.2
	No	1707	95.8
Do you have a family member who has cancer other than breast or cervical cancer?			
	Yes	470	26.4
	No	1312	73.6
Is it possible for breast cancer to be detected early?			
	Yes	1356	76.1
	No	29	1.6
	No idea	397	22.3
How can breast cancer be diagnosed early?			
	Self-examination	451	25.3
	Examination by physician	673	37.8
	Mammography	396	22.2
	Breast ultrasound	126	7.1
	No idea	226	12.7
Do you do self-breast examination?			
	Yes	908	51.0
	No	874	49.0
Have you received a doctor’s examination before?			
	Yes	585	32.8
	No	1197	67.2
Have you ever received a mammogram before? (40 years or older participants)			
	Yes	463	38.0
	No	754	62.0
How often and at what age should mammography be done?			
	Once a year after age 30	128	7.2
	Once every two years after age 30	77	4.3
	Once a year after age 40	424	23.8
	Once every two years after age 40	147	8.2
	No idea	1006	56.5

Regarding the questions about cervical cancer, four out of five women (80.4%) had received gynaecological examinations. Most of the women who had not received gynaecological examinations did not see such examinations as necessary. One in three (31.6%) women did not know it was possible to detect cervical cancer early, and two in three (64.5%) women had not previously received a Pap smear test. Only 4.9% of women knew (correctly) that the Pap smear test should be done once every 5 years, which is the Turkish standard. The majority (84.2%) of women had not heard about the cervical cancer vaccine, while 62.0% would have liked to receive such a vaccination (Table 4).

**Table 4:** Knowledge and practices regarding cervical cancer.

Question		N	%
Have you ever had a gynaecological examination before?			
	Yes	1433	80.4
	No	349	19.6
Why have you not had a gynaecological examination?			
	I did not see it as necessary	239	68.5
	I had no time	12	3.4
	Doctor did not advise me	7	2.0
	I was embarrassed	16	4.6
	Other	27	7.7
	No response	48	13.8
Have you heard about the pap smear test?			
	Yes	669	37.5
	No	1113	62.5
Is it possible for cervical cancer to be recognized early?			
	Yes	1218	68.4
	No	44	2.5
	No idea	520	29.1
Have you received a pap smear test before?			
	Yes	633	35.5
	No	1149	64.5
How often should the pap smear test for cervical cancer be done?			
	Every year	391	21.9
	Once every 2 years	139	7.8
	Once every 5 years	87	4.9
	No idea	1165	65.4
Have you heard about the cervical cancer vaccine?			
	Yes	281	15.8
	No	1501	84.2
Would you like to receive a cervical cancer vaccination?			
	Yes	1104	62.0
	No	263	14.8
	No idea	415	23.2

## Discussion

With the aging of the global population, the incidence of breast and gynaecological cancers is increasing. According to international cancer records, all cancers are more common among older people than among younger people [15,16]. Compared with men, women are particularly susceptible to more chronic diseases and cancers as they age, as they enter the postmenopausal period. Breast cancer and cervical cancer are among the most common causes of cancer deaths in women over forty years of age [17].

A total of 41.8% of the women who participated in the survey had an individual in their family who had a cancer diagnosis. The rate of cancer in Turkey varies between 7.8% and 47.6% [18–24].

Early diagnosis of cancer allows the disease to be identified prior to the onset of disease symptoms. Early diagnosis is aimed at reducing deaths from cancer, increasing the likelihood and success of treatment, and prolonging the survival period [1]. According to the literature, breast cancer can be diagnosed early by self breast examination (SBE), clinical breast examination (CBE) and mammography [25]. The majority (62.0%) of the women who participated in this study had never undergone mammography. In two different studies, the proportions of those who had never undergone mammography were found to be 55.1% [19] and 89.4% [24]. Two studies of midwives and nurses–populations who are expected to have positive health behaviours–found that 87.3% and 58.3%, respectively, had not undergone mammography [21,23]. The very low frequency of mammography in these studies, which were performed on healthcare personnel, can be explained by the fact that the study group was younger than the age group recommended to receive mammography.

It is noteworthy that the rates of SBE are found to be low in studies conducted among diverse women in Turkey. In one study, no women between the ages of 20–60 regularly performed SBE [26]. In another study, only 10% of women were found to be performing SBE occasionally [27]. In our study, 51.0% of participants performed SBE regularly. This result can be attributed to the training provided by the Cancer Screening and Education Center of the region.

Among participating women, the rate of never having had a Pap smear was found to be 64.5%. Similar findings were obtained in other studies conducted in Turkey [24,28]. This rate was 25.0% in a study conducted among American women living in rural areas [29], and it was 26.0% among Korean women living in the United States [30]. Countries differ in how frequently they recommend cervical cancer screening. In Turkey, the recommendation is for Pap smear testing at five-year intervals. Only 4.9% of women in our study correctly knew that recommendation.

In a study conducted among the general population in Turkey, the frequency of having heard about the HPV vaccine was 62.2% [31]. In another study of patients who visited a gynaecology polyclinic, the frequency was 44.6% [32]. Although 84.2% of participants in the present study had not heard about the HPV vaccine, 62.0% seemed eager to receive it, which suggests that women are willing to take precautions for cancer if they are properly directed by health professionals.

This study finding showed that even if there is an increase in the level of knowledge about cancer screening compared to previous studies in Turkey, this is certainly not enough. While the number of participants in the previous rural area studies was low, this study conducted a large survey around the district and provided more accurate information to the literature.

## Conclusion

It has been determined that women do not have sufficient knowledge of cancer diagnosis, early cancer diagnosis, and cancer screening. As a result of this study, awareness of cancer was increased by providing training about risk factors, indicators, prevention, early diagnosis and screening for breast, cervical and colon cancers, which are included in our national cancer screening program.

During the planning, implementation and evaluation of health policies and health services at the national and regional levels, training programs should be developed to raise awareness of cancer, with a primary focus on women. Increasing women’s awareness of cancer prevention, and increasing adaptation to early diagnosis methods, should aim to promote behavioural changes that will spread from the family to the collective. The most likely place to promote cancer prevention and the use of early diagnosis methods is as close to women’s homes as possible. This could be accomplished in the community by continuing to promote education about cancer diagnosis, early diagnosis, screening and prevention.

## Supporting information

S1 Data SPSS Dataset. (SAV)

S1 File Questionnaire in Turkish. (DOCX)

S2 File Questionnaire in English. (DOCX)
