# Supplementary material for: Knowledge, attitudes and behaviours of women regarding breast and cervical cancer in Malatya, Turkey
Source: PLoS One. 2017 Nov 28;12(11):e0188571. doi: 10.1371/journal.pone.0188571 (PMC5705106; doi:10.1371/journal.pone.0188571)
Supplement: S1 File — (DOCX) [file pone.0188571.s002.docx]

KADINLARIN MEME VE SERVIKS KANSERI KONUSUNDA BILGI, TUTUM VE DAVRANIŞLARI

Bu çalışmada “**Kadınların Meme ve Serviks Kanseri Konusunda Bilgi, Tutum ve Davranışları**” amaçlanmıştır. Değerli katılımcılar; sorulara vermiş olduğunuz cevaplar çalışmamıza ışık tutacak ve bu konunun daha iyi anlaşılması konusunda çok faydalı olacaktır. Bu çalışmaya katılım tamamen gönüllülük esasına dayalı olup, veriler bilimsel amaç dışında kullanılmayacaktır. Katılımcılar ad ve soyadlarını kesinlikle belirtmeyeceklerdir. Bu araştırma için siz herhangi bir parasal sorumluluk altına girmiyorsunuz ve size de bir ödeme yapılmayacaktır. Araştırmaya katıldığınız için teşekkür ederiz.

**1. Yaş:** ____________

**2.Eğitim durumunuz nedir? …………………………..1)** okur yazar değil **2)** okur yazar **3)** ilkokul **4)** ortaokul **5)** lise **6)** üniversite

**3.Mesleğiniz nedir?** ……………………………………**1)** Ev hanımı **2)** Memur **3)** İşçi **4)** Emekli memur  **5)** Emekli işçi **6)**Serbest meslek

**4.Medeni durumunuz nedir?.........................................1)** Evli **2)**Bekar **3)**Dul (**eşi ölmüş**) **4)** Boşanmış

**5. Evli iseniz kaç yaşında evlendiniz?_______________**

**6.Yaşadığınız yer:………………………………………1)**İl **2)** İlçe **3)**Köy

**7.Ekonomik durumunuz ?..............................................1)**Kötü **2)** Orta **3)**İyi

**8. Sağlık Güvenceniz var mı? …………………………1)** Var  **2)** Yok

**9. İlk adet yaşınız:________________**

**10. Adetten kesilme yaşınız:_______**

**11. İlk doğum yapma yaşınız:_______**

**12. Gebelik sayısı…………………………...…………..0) 0 1) 1 2) 2 3) 3 4) 4 5) 5 6) 6 7) 7 8) diğer (______)**

**13. Düşük sayısı………………………………………… 0) 0 1) 1 2) 2 3) 3 4) 4 5) 5 6) 6 7) 7 8) diğer (______)**

**14. Canlı Doğum sayısı…… …………….………...….. 0) 0 1) 1 2) 2 3) 3 4) 4 5) 5 6) 6 7) 7 8) diğer (______)**

**15. Yaşayan çocuk sayısı…………………………...….. 0) 0 1) 1 2) 2 3) 3 4) 4 5) 5 6) 6 7) 7 8) diğer (______)**

**16. Hangi Aile Planlama yöntemini kullanıyorsunuz?.......1)** Kondom  **2)** RİA **3)** Enjeksiyon **4)** HAP **5)**Geri Çekme **6)** Kullanmıyorum

**17. Hayatınız boyunca hiç sigara içtiniz (5 paket) mi?.........1)** Evet **2)** Hayır

**18. Halen sigara içiyor musunuz? 1)** Evet, hergün en az 1 adet 2) Hergün olmamakla birlikte ara sıra **3)** İçiyordum bıraktım **4**) Hiç içmedim

***(Evet ise içilen miktar:____________ adet/gün veya_______________adet/hafta***)

**19.** **Sigara içmeye başladığınızda kaç yaşındaydınız?**________

**20. Meme kanseri ve Rahim ağzı kanserinin erken tanısı için yapılan taramaların ücretsiz olduğunu biliyor musunuz?**.............**1)**Evet **2)**Hayır

**21. Ailede “meme kanseri” tanısı alan akrabanız var mı?.............. 1)**Var **2)**Yok

**22. 21. Soruya cevabınız “Var” ise hangi akrabanız meme kanseri tanısı aldı? 1)**Annem **2)**Ablam/Kardeşim **3)**Teyzem **4)**Halam **5)**Diğer(_____________)

**23. Aile “Rahim Ağzı Kanseri” tanısı alan akrabanız var mı?...... 1)**Var **2)**Yok

**24. 23. Soruya cevabınız “Var” ise hangi akrabanız “Rahim ağzı kanseri” tanısı aldı? 1)**Annem **2)**Ablam/Kardeşim **3)**Teyzem **4)**Halam **5)**Diğer(______)

**25. Ailenizde meme ve Rahim ağzı kanseri DIŞINDA kanser tanısı alan kimse var mı? (VARSA KİM-HANGİ KANSER?)**

**1) Var (KİM-HANGİ KANSER?___________________________----_____________________________________) 2)Yok**

**26. Sizce meme kanserinin erken dönemde tanınması mümkün müdür?........................... 1)** Evet **2)** Hayır **3)** Fikrim yok

**27. 26. Soruya cevabınız EVET ise meme kanserine nasıl erken tanı konulabilir? *(Birden fazla şık işaretleyebilirsiniz)***

**1)**Kendi kendine meme muayenesi **2)**Hekimin muayenesi **3)**Mamografi **4)**Meme ultrasonu **5)**Bilmiyorum

**28 Erken tanı konulursa meme kanseri tedavi edilebilir mi?.............................................1)** Evet **2)** Hayır **3)** Fikrim yok

**29. Kendi kendinize meme muayenesi yapmasını biliyor musunuz? (BİLİYORSANIZ KİMDEN ÖĞRENDİNİZ?)**

**1)** Evet (**KİMDEN ÖĞRENDİNİZ?( 1- Aile Hekimim 2-Diğer doktorlardan 3- İnternet veya Televizyondan 4- Bilen bir tanıdığımdan)** **2)** Hayır

**30. Kendi kendinize meme muayenesi yapıyor musunuz?..................................................1)**Evet **2)** Hayır

**31. Sizce kendi kendine meme muayenesi ne sıklıkla yapılmalıdır?...................................1)**Hergün **2)** Haftada bir **3)** Ayda bir **4)**Altı ayda bir  **5)** Yılda bir

**32. Sizce hangi dönemde kendi kendine meme muayenesi yapılmalıdır?........................1)** Her zaman **2)** Adetten önce  **3)** Adet döneminde **4)** Adetten sonra

**33. Sizce aşağıdaki belirtilerden hangisi ya da hangileri meme kanseri belirtisi olabilir? *(Birden fazla şık işaretleyebilirsiniz)***

**1)** Memede şişlik ya da kitle **2)** Meme başından gelen kanlı akıntı **3)** Meme başının içe çekilmesi **4)** Memede yara

**5)** Koltuk altında şişlik ya da kitle **6)** Memede renk değişikliği **7)** Memede ağrı **8)** Bilmiyorum

**34.** **Daha önce “doktora” meme muayenesi yaptırdınız mı?**.............................................**1)**Evet **2)** Hayır

**35.** **Cevabınız evet ise meme muayenenizi kim yaptı?..................1)**Aile hekimi **2)**Genel cerrahi uzmanı  **3)**Kadın doğum uzmanı **4**)Diğer (_____________)

**36. Doktora meme muayenesi yaptırmadıysanız nedeni nedir?  *(Birden fazla şık işaretleyebilirsiniz)***

**1)**Gerekli görmedim **2)** Zamanım yoktu **3)**Hekim önermedi **4)**Utandım  **5)Diğer (_________________)**

**37. “Doktora” ne sıklıkla meme muayenesi yaptırılmalıdır? 1)** Gerek yok 2**)** Ayda bir **3)** Altı ayda bir **4)** Yılda bir **5)** Şikayetim olunca **6)** Fikrim yok

**38. Daha önce hiç mamografi çektirdiniz mi?........................................................................** **1)** Evet **2)** Hayır

**39. Sizce mamografi ne sıklıkla ve kaç yaşından sonra çektirilmeli?**

**1) 30** yaşında sonra **yılda bir 2) 30** yaşından sonra **iki yılda bir** **3) 40** yaşından **sonra yılda bir** **4) 40** yaşından sonra **iki yılda bir** 5) Fikrim yok

**40. Kendi kendinize meme muayenesi yaparken hiç kitle tespit ettiniz mi?..........................1)** Evet **2)** Hayır

**41. Eğer kitle tespit edip doktora başvurduysanız sonuç ne geldi?.................1)**Kötü huylu kitle **2)**İyi huylu kitle **3)**Kitle tespit edilmedi

**42. Daha önce jinekolojik muayene oldunuz mu?....................................................................1)** Evet **2)** Hayır

**43. Cevabınız hayır ise neden?** **1)**Gerekli görmedim **2)** Zamanım yoktu **3)**Hekim önermedi **4)**Utandım **5)Diğer (______________)**

**44. Jinekolojik muayeneye gitme sıklığınız nedir**  **1)** Hastalığın verdiği sıkıntıya dayanamadığımda **2)** Herhangi bir şikayetim olunca **3)** Düzenli aralıklarla

**45. Daha önce cinsel yolla bulaşan hastalığınız oldu mu?......................................................... 1)** Evet **2)** Hayır

**46. Cevabınız evet ise hangi hastalık?_______________________________________________________________________________________________**

**47. Pap smear testini duydunuz mu?...........................................................................................** **1)** Evet **2)** Hayır

**48. Sizce Rahim Ağzı Kanserinin erken dönemde tanınması mümkün müdür?.................... 1)** Evet **2)** Hayır **3)** Fikrim yok

**49. 48. Soruya cevabınız EVET ise Rahim Ağzı Kanserine nasıl erken tanı konulabilir?......1)**Hekimin muayenesi **2)** Pap Smear **3)**Ultrason **4)** Fikrim yok

**50. Erken tanı konulursa Rahim Ağzı Kanseri tedavi edilebilir mi?........................................1)** Evet **2)** Hayır **3)** Fikrim yok

**51. Pap smear testi hangi hastalığın tanısı için yapılmaktadır? ……………. 1)** Rahim ağzı kanseri **2)** Meme kanseri **3)** Barsak kanseri 4) Bilmiyorum

**52. Daha önce pap smear testi yaptırdınız mı? (SAYISINI İŞARETLEYİNİZ 1)** Evet (**Kaç kez?__1) 2) 3) 4) 5) 6) 7) )** **2)** Hayır

**53. Cevabınız evet ise smear testi sonucunuz ne geldi?................... 1) N**ormal / Sağlıklı **2)** Servisit **3)** HPV **4) Diğer_________** **5)**Bilmiyorum

**54. Rahim ağzı kanseri taraması için taraması için Pap smear testi ne sıklıkta yapılmalıdır?... 1)** Her yıl **2)** 2 yılda bir **3)** 5 yılda bir **4)**bilmiyorum

**55. Rahim ağzı kanseri yönünden riskli olduğunuzu düşünüyor musunuz? …………….. 1)** Evet **2)** Hayır **3)** Fikrim yok

**56. 55. Soruya cevabınız evet ise neden riskli olduğunuzu düşünüyorsunuz?______________________________________________________________**

**57. Rahim ağzı kanseri aşısını duydunuz mu? ……………………………………………… 1)** Evet **2)** Hayır

**58. Sizce Rahim ağzı kanseri aşısını yaptırmak rahim ağzı kanserinden korur mu?...........1)** Evet **2)** Hayır **3)** Fikrim yok

**59. Birden fazla cinsel partner Rahim Ağzı kanseri nedenleri arasında mıdır?................... 1)** Evet **2)** Hayır **3)** Fikrim yok

**60. Kondom kullanımı rahim ağzı kanserinden korur mu?....................................................1)** Evet **2)** Hayır **3)** Fikrim yok

**61. Rahim ağzı kanseri aşısını yaptırmak ister misiniz?..........................................................1)** Evet **2)** Hayır **3)** Fikrim yok

**62. Cevabınız hayır ise nedeni nedir? ______________________________________________________________________________________________**
