# Supplementary material for: Knowledge, attitudes and behaviours of women regarding breast and cervical cancer in Malatya, Turkey
Source: PLoS One. 2017 Nov 28;12(11):e0188571. doi: 10.1371/journal.pone.0188571 (PMC5705106; doi:10.1371/journal.pone.0188571)
Supplement: S2 File — (DOCX) [file pone.0188571.s003.docx]

Knowledge, Attitudes And Behaviours Of Women Regarding Breast And Cervical Cancer

(original is Turkish)

In this study, "Knowledge, Attitude and Behaviors of Women on Breast and Cervical Cancer" were aimed. Dear participants; the answers you have given to the questions will shed light on our work and it will be very useful for a better understanding of this topic. Participation in this work is entirely voluntary, and the data will not be used for purposes other than scientific. Participants will not necessarily give their names and surnames. You are not under any financial responsibility for this research and no payment will be made to you. Thank you for participating in the investigation.

**1. Age:** ____________

**2.** **What is your education status? …………………………..** 1) not literate 2) literate 3) primary school 4) middle school 5) high school 6) university

**3.** **What is your job?** …………………………………… 1) Housewife 2) Officer 3) Worker 4) Retired employee 5) Retired worker 6) Self employed

**4.** **What is your marrital status?.........................................** 1) Married 2) Single 3) Widowed 4) Divorced

**5. How old were you married when you married? _______________**

**6.** **Your place of residence:………………………………………** 1) Province 2) District 3) Village

**7.** **Your economic situation?..............................................** 1) Bad 2) Moderate 3) Good

**8. Do you have health insurance? …………………………** 1) Yes 2) No

**9. Age of first menstruation:________________**

**10. Age of end of menstruation:_______**

**11. Age when first childbirth:_______**

**12. Number of pregnancies…………………………...…………..**0) 0 1) 1 2) 2 3) 3 4) 4 5) 5 6) 6 7) 7 8) other (______)

**13. Number of miscarriage…………………………………………** 0) 0 1) 1 2) 2 3) 3 4) 4 5) 5 6) 6 7) 7 8) other (______)

**14. Number of live births…… …………….………...…..** 0) 0 1) 1 2) 2 3) 3 4) 4 5) 5 6) 6 7) 7 8) other (______)

**15. Number of living children …………………………...…..** 0) 0 1) 1 2) 2 3) 3 4) 4 5) 5 6) 6 7) 7 8) other (______)

**16. Which Family Planning method do you use** 1) Condom 2) IUD 3) Injection 4) Pills 5) Withdrawal 6) I do not use

**17. Have you ever smoked (5 packs) during your life?** 1) Yes 2) No

**18. Do you smoke now?** 1) Yes, every day at least 1 2) Occasional with not everyday 3) I quit smoking 4) I did not smoke *(If yes, the amount is ____________ units / day or _______________ units / week)*

**19.** **How old were you when you started smoking?** ______

**20. Do you know that scans for early detection of breast cancer and cervical cancer are free?** 1)Yes 2)No

**21. Do you have a family member who has a "breast cancer" in the family?..............** 1)Yes 2)No

**22. If you answered "Yes" to question 21, which of your relatives has breast cancer?** 1) My mother 2) My brother / sister 3) Maternal aunt 4) Paternal aunt 5) Other (_____________)

**23. Do you have a family member who is diagnosed as Family "Cervical Cancer"?......** 1)Yes 2)No

**24. If your answer to question 23 is "Yes", which of your relatives has the word "cervix cancer"?** 1) My mother 2) My brother / sister 3) Maternal aunt 4) Paternal aunt 5) Other (_____________)

**25. Is there anyone who is diagnosed with cancer other than breast cancer and cervical cancer? (WHO - WHICH CANCER?)**

1) Yes (WHO-WHICH CANCER?___________________________----_____________________________________) 2)No

**26. Is it possible for you to have early diagnosis of breast cancer?...........................** 1) Yes 2) No 3) No idea

**27. If the answer to question 26 is YES, how can breast cancer be diagnosed early? (You can mark multiple answers)**

1) Breast self examination 2) Doctor's examination 3) Mammography 4) Breast ultrasonics 5) I do not know

**28 Can breast cancer be treated if it is diagnosed early?** 1) Yes 2) No 3) No idea

**29. Do you know how to do breast self-examination yourself? (WHO DID YOU LEARN FROM?)**

**1)** Yes (WHO THOUGHT?) (1- Family Physician 2-From Other Doctors 3- Internet or Television 4- From a Knowing Person)**2)** No

**30. Do you do self breast examination?..................................................1)**Yes **2)** No

**31. How often should you do your own breast examination?...................................** 1) Daily 2 ) Once a week 3) Once a month 4) Every six months 5) Once a year

**32.** **In which period of time should you self-examine your breast??........................** 1) Always 2) Before menstruation 3) In menstruation 4) After the menstruation

**33. Which of the following symptoms may or may not be a sign of breast cancer? (You can mark multiple answers)**

1) Breast swelling or mass 2) Bleed current from the nipple 3) Absorbtion of nipple head 4) Wound in breast

5) Swelling or mass under armpit 6) Color change of breast 7) Breast pain 8) I do not know

**34.** **Have you ever had a "doctor" breast examination?**.............................................1)Yes 2) No

**35.** **If yes, who did your breast examination?..................** 1) Family physician 2) General surgeon 3) Gynecologist 4) Other (_____________)

**36. What is the reason if you did not have a doctor's breast examination? (You can mark multiple answers)**

1) I did not see it necessary 2) I did not have the time 3) The physician did not recommend 4) I was ashamed 5) Other (_________________)

**37. “How often should the "breast examination by doctor" be performed?** 1) No need 2) Once a month 3) Every six months 4) Once a year 5) After complaints 6) No idea

**38. Have you ever taken a mammogram before?........................................................................** **1)** Yes **2)** No

**39. How often and after how many years should your mammogram be taken?**

1) once a year after 30 years 2) every two years after 30 years 3) once a year after 40 years 4) every two years after 40 years 5) No idea

**40. Did you identify any masses while performing breast examination yourself?..........................1)** Yes **2)** No

**41. If you detect a mass and refer to a doctor, what is the result?.................** 1) Malignant mass 2) Benign mass 3) Mass not detected

**42. Did you have a gynecological examination before?....................................................................1)** Yes **2)** No

**43. If the answer is no, why not?**

1) I did not see it necessary 2) I did not have time 3) The physician did not recommend 4) I was embarrassed 5) Other (______________)

**44. What is your frequency of going to the gynecologic examination ?**

1) When I can not stand the trouble of the disease 2) When any complaints 3) Regular intervals

**45. Have you ever had sexually transmitted disease?......................................................... 1)** Yes **2)** No

**46. ​​If yes, what disease?_______________________________________________________________________________________________**

**47. Did you hear Pap smear test?...........................................................................................** **1)** Yes **2)** No

**48. Is it possible for to have early diagnosis of cervical cancer?.................... 1)** Yes **2)** No **3)** No idea

**49. If YES to question 48, how can you diagnose Cervical Cancer early?** 1) Doctor's examination 2) Pap Smear 3) Ultrasound 4) No idea

**50. Can cervical cancer be treated if early diagnosis is made?........................................1)** Yes **2)** No **3)** No idea

**51. What disease is the Pap smear test done for?…………….** 1) Cervical cancer 2) Breast cancer 3) Bowel cancer 4) I do not know

**52. Did you have a pap smear test before? (MARK YOUR NUMBER)** Yes (How many times? **1) 2) 3) 4) 5) 6) 7)**  **2)** No

**53. If yes, what happened as a result of the smear test?...................** 1) Normal / healthy 2) Cervicitis 3) HPV 4) Other _________ 5) I do not know

**54. How often should the Pap smear test be done to scan the cervix for cancer screening?...** 1) Every year 2) Every 2 years 3) Every 5 years 4) I do not know

**55. Do you think you are ​​risky for cervical cancer?……………..** 1) Yes 2) No 3) No idea

**56. If yes to question 55, why do you think you are at risk?______________________________________________________________**

**57. Have you heard of cervical cancer vaccination? ……………………………………………… 1)** Yes **2)** No

**58. Do you take cervical cancer vaccine to protect you from cervical cancer?1)** Yes **2)** No **3)** No idea

**59. Are there multiple sexual partners among the causes of cancer of the cervix?................... 1)** Yes **2)** No **3)** No idea

**60. Do condom use protect from cervical cancer?.................................................... 1)** Yes **2)** No **3)** No idea

**61. Do you want to have cervical cancer vaccination??.......................................................... 1)** Yes **2)** No **3)** No idea

**62. What is the reason if your answer is no? ______________________________________________________________________________________________**
